# Providing behavioral workforce development technical assistance during COVID-19: adjustments and needs

**DOI:** 10.1093/tbm/ibab097

**Published:** 2021-08-19

**Authors:** Cross-Technology Transfer Center (TTC) Workgroup on Virtual Learning, Sara Becker, Abby Kisicki, Michael Chaple, Thomas E Freese, Heather Gotham, Rachelle Greller, Holly Hagle, Maxine Henry, Laurie Krom, Rosemarie Martin, Kristen Powell, Nancy Roget, Isa I Velez-Echevarria, Ruth Yáñez, Todd Molfenter

**Affiliations:** University of Wisconsin–Madison, Madison, WI, USA

**Keywords:** COVID-19, Behavioral health, Workforce, Telehealth

## Abstract

COVID-19 social distancing policies have triggered a historic shift in the delivery of behavioral health prevention and treatment services. Among the first responders to this monumental workforce development challenge were the Substance Abuse and Mental Health Services Administration-funded Technology Transfer Centers (TTCs), which are charged with building the behavioral health workforce’s capacity to provide evidence-based prevention, treatment, and recovery services. TTCs documented unprecedented attendance at their events in the early months of the pandemic. This study applied content analysis to identify the most common COVID-related technical assistance (TA) topics and examine attendance by topic from March to July 2020. Across 393 events, TA topics explicitly related to COVID-19 encompassed eight emergent themes: (a) delivering services via telehealth, (b) providing support and services to behavioral health consumers, (c) promoting workforce self-care, (d) understanding new laws/policies, (e) delivering evidence-based practices, (f) advancing racial equity, (g) offering networking spaces, and (h) altering organizational management and communication infrastructure. The most heavily attended events focused on the TA themes “Advancing Racial Equity” (average = 352) and “Telehealth Service Delivery” (average = 271). There was a documented shift from more intensive TA to briefer, more targeted TA provision. The TTCs rapidly virtualized training and TA offerings to address workforce needs and serve as a model for providing remote workforce development support during the COVID-19 pandemic and future national crises.

**Implications**

**Practice:** Across 393 training and technical assistance (TA) events in the first six months of the COVID-19 pandemic, the Substance Abuse and Mental Health Administration’s Technology Transfer Centers (TTCs) addressed eight main themes: (a) delivering services via telehealth, (b) providing support and services to behavioral health consumers during COVID-19, (c) promoting workforce self-care, (d) understanding new laws/policies, (e) delivering evidence-based practices, (f) advancing racial equity, (g) offering networking spaces, and (h) altering organizational management and communication infrastructure during COVID-19. The behavioral health workforce needed training in these areas to provide high-quality services within the context of the COVID-19 pandemic.

**Policy:** The TTCs’ ability to innovate training and TA rapidly in response to workforce needs can serve as a model for providing workforce development TA during the COVID-19 pandemic and future national crises.

**Research:** Future studies should continue to review the behavioral health workforce’s training and TA needs during and following the COVID-19 pandemic.

COVID-19 social distancing policies triggered a historic change in the delivery of behavioral health prevention and treatment services [1–5]. Rapid shifts to telemedicine and remote strategies (e.g., individual and group therapy, medication management, and school-wide prevention interventions) are becoming system-altering innovations, similar to the use of pharmacotherapies for addiction and mental health disorders [6, 7]. Prior system-altering innovations transpired over years and decades, whereas COVID-19-induced changes happened in a matter of days and weeks.

Among the first responders to this monumental workforce development challenge were the Substance Abuse and Mental Health Services Administration-funded Technology Transfer Centers (TTCs). The TTCs constitute three distinct networks with different foci but with shared infrastructure and operational procedures: Addiction TTCs, Mental Health TTCs, and Prevention TTCs. The TTCs, which serve all 50 states and U.S. territories, are charged with building the behavioral health workforce’s capacity to provide evidence-based prevention, treatment, and recovery services by delivering timely and culturally responsive training and technical assistance (TA) tailored to local needs [8]. Each TTC Network consists of 10 Regional Centers, 2 National Population-Specific Centers, and a Network Coordinating Office (total of 39 centers). Through September 2020, five international HIV TTCs served Ukraine, South Africa, Vietnam, and Southeast Asia.

In response to the rapid virtualization of services, the TTCs had to immediately assess what types of TA to offer and how to deliver them. Before COVID-19, TA delivery included in-person and virtual offerings in three categories: Basic, Targeted, and Intensive [9]. Basic TA provides information dissemination and consists of brief consultation, mass mailings, publications, e-newsletters, websites, social media, and single-event webinars. Targeted TA enhances practitioners’ readiness and builds the capacity to implement evidence-based practices. Targeted TA typically consists of online courses, webinar series, communities of practice, and short-term training. Intensive TA supports full incorporation of innovation or practice into real-world settings that requires changes in policies, practices, and system functioning. Changes are ultimately driven by an implementation plan that reflects mutually agreed-upon goals, roles, and responsibilities between the TA provider and recipient [10]. COVID-19 lockdown and social distancing requirements prohibited face-to-face training and required virtual delivery of all Basic, Targeted, and Intensive TA.

This study leveraged both quantitative survey data and qualitative content analysis to investigate how the nature of TA provided by the TTCs changed during the COVID-19 pandemic. The analyses addressed two specific aims. First, we examined how TTC Directors and their teams adjusted TA provision, including perceived benefits and challenges of virtual TA delivery during the pandemic. Second, we identified the most common TA topics related to COVID-19 during the early months of the pandemic (March–July 2020), as well as the number of participants that attended events in each topic category. The overarching goal of this analysis was to discern how this large national federally funded network of behavioral health training and TA centers shifted from in-person to entirely virtual service delivery, as well as the most popular COVID-related training and TA topics, to identify best practices for the provision of remote workforce development support in future national crises.

## METHODS

### Study context

Data were compiled from the TTCs in the USA and the international TTCs serving Ukraine, South Africa, Vietnam, and Southeast Asia. This study analyzed events occurring in the early months of the pandemic, between March and July 2020.

### Data sources

The study used two data sources and integrated both qualitative and quantitative data. First, a workgroup surveyed TTC regional (*n* = 30), national (*n* = 9), and international grantees (*n* = 5) to document the delivery of TA services. The TTC survey was completed by the TTC Directors on behalf of their Center in May 2020; TTC Directors were encouraged to confer with their full team to answer questions. The survey collected quantitative information on the platform used to provide virtual trainings (e.g., Zoom and Microsoft Teams) and asked each TTC Center to self-report the estimated percentage of TA provision by category (e.g., Basic, Targeted, and Intensive) over two time periods: (a) before the COVID-19 pandemic and (b) during the COVID-19 pandemic. The survey also asked a series of open-ended qualitative questions on the perceived benefits and challenges of moving to 100% virtual training during COVID-19 (e.g., What do you see as the benefits of the shift to fully virtual TA for your TTC? What do you see as the challenges of the shift to fully virtual TA for your TTC?). The second data source was the TTC events database which includes the TA title, description, category, and number of people who attended each TA event. Only those events specifically flagged as COVID-19-related by each TTC from March to July 2020 were pulled for analysis.

### Data analysis

Qualitative content analysis was used to identify the most common benefits and challenges of providing TA in an entirely virtual format, based on open-ended responses to the TTC survey. In addition, content analysis was used to identify the most common TA topics during the initial COVID-19 period, based on titles in the events database.

Content analysis used a step-wise, pragmatic approach. For the open-ended survey questions, two Ph.D. researchers read all responses individually, met to group quotes with similar meaning, created a list of themes, and tabulated frequency counts of the quotes aligning with each theme. The researchers also extracted illustrative quotes demonstrating each theme. This streamlined content analysis process was used due to the relatively small number of quotes (*n* = 42 responses). For the event data, a more dynamic, team-based approach was used to analyze the larger number of events (*n* = 393) adequately. The approach followed steps similar to those outlined by Bicudo de Faria-Schützer and colleagues [11]. Four TTC team members (the same two who analyzed the leadership survey and two BA-level staff) each read through the list of 393 events to gain holistic impressions (e.g., “free-floating reading”). The four staff members then met to group events with similar themes (e.g., constructed units of analysis), define an initial set of codes, and develop a detailed coding dictionary (e.g., identified cores of the meaning). The four coders’ consensus rated 10% of the dataset and met to discuss initial areas of agreement and disagreement (e.g., discussion of the topics). After in-depth discussion, the remaining events were each double-coded by two independent raters. The entire team of four coders met to review ratings and agree upon final consensus code assignments (e.g., advancing validity). Each event was assigned a maximum of two TA themes to simplify and facilitate the consensus coding process.

Quantitative analysis consisting of basic descriptive statistics was used to quantify the percentage of Basic, Targeted, and Intensive TA provided before and during the period of COVID-19 social distancing, based on each TTC’s report. Additional quantitative analyses were run to calculate the average number of participants for each TA event in each thematic category.

## RESULTS

There was a 93% response rate from the TTCs (*n* = 42) on the survey, and 393 COVID-19 related TA events occurred from March to July 2020.

### TTC survey: change in TA provision

As shown in Table 1, survey data revealed that virtual TA increased from 43.3% pre-COVID-19 to 100% during COVID-19 based on the TTC report. Concurrent with this change, virtual TA moved from a mix of voice only, voice and slides, and video, to video-only platforms. Of the TTCs who completed the survey, 80% used Zoom, 25% Adobe, 7.5% WebEx, 2.5% Microsoft Teams, and 2.5% GoToMeeting to deliver TA. Several TTCs were reported using multiple platforms. In response to workforce needs to acquire new skills, TTCs increased Targeted TA by 14.5% and reduced the proportion of Intensive TA by 14.2%.

**Table 1:** | Types of technical assistance (TA) offered by the Technology Transfer Centers based on Director report

TA type	% Virtual before COVID-19 (SD)	% Virtual during COVID-19 (SD)	Difference (SD)	% TA type before COVID-19 (SD)	% TA type during COVID-19 (SD)	Difference
Basic TA	49.7% (31.8)	100%	50.3%	40.5% (20.5)	43.2%	+2.7%
Targeted TA	41.1% (28.7)	100%	58.9%	38.0% (16.8)	52.5%	+14.5%
Intensive TA	41.6% (33.3)	100%	58.4%	18.5% (12.5)	4.3%	−14.2%
**Overall**	43.3% (29.9)	100%	56.7%	–	–	–

*SD* standard deviation.

In open-ended questions in the May 2020 survey, TTCs reported five key challenges when adapting TA to meet urgent workforce needs: difficulty engaging stakeholders, inadequate technology infrastructure, insufficient technical skills, demands on staff time, and security concerns. The most common perceived challenge was the difficulty engaging stakeholders virtually (*n* = 13), with multiple TTCs citing difficulties with “relationship development,” “building trust,” “engaging attendees,” and “experiential learning” in a virtual environment. The next most common concern was insufficient technology infrastructure (*n* = 10), particularly in regions with large rural communities. One TTC noted “large sections of our workforce do not have the internet bandwidth or equipment to fully engage in virtual TA,” and another noted that some communities were “excluded from full participation” due to “lack of broadband [and] lack of financial resources to purchase technology.” Other key concerns included the technical skills needed to adjust to the “technology learning curve” (*n* = 9), increased demands on staff time (*n* = 4), and perceived security issues with virtual platforms (*n* = 3).

There were also five key benefits of virtual TA cited by the TTCs: expanded reach, improved efficiency, decreased cost, increased staff productivity, and increased cross-TTC collaboration. Increased reach was the most commonly cited benefit (*n* = 17). One TTC respondent noted, “Size of our audience has increased, some people can access content more easily virtually and have been given time for professional development,” and another indicated, “Basic/foundational training delivered via virtual means increased access to rural and frontier participants.” In addition, multiple TTCs noted greater efficiency in terms of time savings (*n* = 14) and reduced costs (*n* = 9). For instance, one respondent noted, “Delivering remote TA activities costs less than in-person events due to savings associated with travel, shipping of materials, and staff time spent traveling,” and others described virtual training as “more efficient” and “more affordable.” Other benefits included increased “productivity of full-time staff” and increased opportunities to “collaborate” and “coordinate more” with other TTCs.

### TA events database: TA themes

For TA subject themes and topics of interest, events entered in the TA Events database were coded to identify the most common COVID-related TA topics from March to July 2020. Table 2 defines the emergent themes with example events and the average number of participants per theme. 

**Table 2:** | Technical assistance (TA) thematic codes from 393 unique events

TA code	Definition of code	Coded events (*n*)	Average participants per event (*n*)	Example event title (*n* = number of participants)
Racial Equity	Promote cultural competency and racial equity	28	352	Strategic Discussion: Health Disparities and the Impact of COVID-19 on African American and Black Communities (*n* = 391)
Telehealth	How to safely interact with patients through telehealth	62	271	Where to Begin: Essential Tips for Using Videoconferencing Services to Deliver SUD Treatment and Recovery Services (*n* = 952)
Support Services	How to support and serve behavioral health consumers experiencing COVID-19-related issues	107	246	Supporting Families of Young Children at Risk for Ongoing Domestic Violence (*n* = 855)
Supportive Self-care	Adopt and provide supportive self-care practices for workforce	92	207	Compassion Fatigue: Managing During the Pandemic with Self-Care Strategies (*n* = 450)
Evidence-Based Practices	How to adapt and/or deliver established Evidence-Based Practices during COVID times	61	174	Clinical Innovations in Telehealth: Telehealth and Cognitive Behavioral Therapy in Psychosis (*n* = 540)
Networking	Networking spaces for workforce and TTCs to learn and have discussions with each other	123	137	Learning From and With Students, Caregivers, Advocates and Systems Leaders: Part 1 Supporting School Mental Health in the Context of Racial Violence Discussion Forum (*n* = 813)
Organizational Management and Communication	Altering organizational management and communication infrastructure practices during COVID-19	55	82	Virtual Series: The Post-COVID-19 Workplace—What Will Work Look Like? (*n* = 259)
Changing Laws & Policies	Understand changing laws/policies during COVID-19	8	51	Guidance on Federal Health Privacy Laws for Behavioral Health Practitioners and Peer Support Specialists for Virtual Service Delivery during COVID-19 (*n* = 68)
Total		536	194	

Each event could be assigned up to two codes.

The most commonly offered TA topics were networking (*n* = 123) and support and services (*n* = 107). Virtual networking events were generally framed as forums to facilitate interactions with other providers around a specific discussion topic (e.g., a discussion forum on how to cope with specific COVID-19-related professional stressors) or specific activity (e.g., coffee chat, mindfulness exercise, and lunch break): these events were offered frequently in the wake of social distancing requirements. Meanwhile, TA events focused on support and services were offered frequently for members of the workforce seeking guidance on how to help their clients address emergent challenges during the COVID-19 pandemic. Of note, events focused on racial equity were not the most frequently offered but were the most highly attended (*n* = 352 on average); these events addressed inequities in behavioral health prevention, treatment, and recovery. Attendance at health equity events was consistently high throughout the COVID-19 reporting period, though notably, the highest attended the event in this category (*n* = 711 participants) was on June 19, a few weeks after the death of George Floyd.

## Discussion

This analysis revealed that the TTCs were able to rapidly adjust to provide training and TA to large numbers of stakeholders in the early months of the COVID-19 pandemic. Content analysis elucidated a number of perceived benefits and challenges of the virtualization of training and TA services. Consistent with research evaluating virtual service provision [12], the perceived benefits included improved reach, increased efficiency, and reduced costs. Many TTCs highlighted higher attendance at virtual events and greater efficiency of service provision. Also, in harmony with prior research [13, 14], perceived challenges included the difficulties making virtual training and TA engaging, the technical skills required, and the demands on staff time. Most concerning, TTC respondents shared that large portions of the workforce did not have the internet bandwidth or equipment to engage in training: such concerns were predominantly articulated by TTCs serving regions with large rural communities. Future work by our team aims to more closely examine the reach of TTC events in rural and underserved communities to evaluate whether the well-documented digital divide [13, 15] could exacerbate disparities in access to training and TA.

Data from this analysis indicate that the behavioral health workforce was seeking TA to help adjust to the delivery of behavioral health services under COVID-19 social distancing safety precautions. There was a marked shift in TA recipients moving away from Intensive TA toward briefer, lighter touch Targeted TA. The low proportion of Intensive TA events provided and attended suggests that transitioning Intensive TA from in-person to virtual programming was problematic for the participants and TTCs. Intensive TA requires a substantial and continuous-time investment from both the TA provider and recipients [10]. In the early days of the COVID-19 pandemic, the behavioral health workforce may have been seeking solutions that could be deployed more rapidly to address their training and TA needs.

It is important to note that many of the TA themes identified in the content analysis of COVID-relevant events, such as providing effective support and services to behavioral health consumers, promoting provider self-care, understanding substance use laws/policies, delivering evidence-based practices, and advancing racial equity were popular TTC TA topics before COVID-19, as evidenced by national curricula in these areas. In response to the pandemic, the TA events’ focus shifted to reflect new stressors, practice delivery models, laws, and regulations. By contrast, topics such as networking, telehealth service delivery, and how to alter organizational management and communication to accommodate virtual delivery were rarely offered before COVID-19; these TA topics emerged in direct response to social distancing requirements. The TTCs’ work in these topic areas can help accelerate the use of telehealth [16] and address COVID-19 related increases in behavioral health disorders and acuity [17, 18].

It is also remarkable that in the early months of the COVID-19 pandemic, TA events focused specifically on social justice, racial equity, and health disparities were not the most frequently offered but were the most heavily attended. The murder of George Floyd, subsequent protests across the country, and the pronounced health disparities highlighted through the differential impact of COVID-19 on communities of color likely increased the behavioral health workforce’s collective interest in these topics [19, 20]. The TTCs had developed considerable TA programming in this area since the Addiction TTC Network’s inception in 1993. However, the culmination of the inequitable impact of COVID-19 and the events of the summer of 2020 appreciably increased the interest in and urgency of developing better practices to address racial equity and social injustice issues.

A primary limitation of this analysis was the reliance on TTC teams’ self-report to estimate the types of TA provided (e.g., Basic, Targeted, and Intensive) before the pandemic, which might have been subject to recall bias. In addition, the reliance on event titles and descriptions to discern specific TA topics might not have fully captured the focus of events. We also focused on those events that TTCs designated as COVID-related to examine TA needs that arose explicitly due to the pandemic; a broader analysis of all TA events might have revealed additional emergent themes.

Notwithstanding these limitations, this survey provides data from a large national network of federally funded TA centers and elucidates emergent areas of TA need during the early days of the COVID-19 pandemic. The TTCs, much like the providers they serve, had to nimbly respond to the changes caused by the COVID-19 pandemic to provide services to a population in need: the behavioral health workforce. The TTCs’ capacity to rapidly virtualize training and TA in response to workforce needs can serve as a model for providing remote workforce development TA during the ongoing COVID-19 pandemic and future national crises. Future research comparing the effectiveness of in-person and virtual TA provision by theme is needed to determine the optimal training and TA provision mix after the pandemic.

**Contributing authors:** Sara Becker, Ph.D., Abby Kisicki, B.A., Michael Chaple, Ph.D., Thomas E. Freese, Ph.D., Heather Gotham, Ph.D., Rachelle Greller, MS, Holly Hagle, Ph.D., Maxine Henry, MSW, MBA, Laurie Krom, M.S., Rosemarie Martin, Ph.D., Kristen Powell, Ph.D., Nancy Roget, M.S., Isa I. Velez-Echevarria, PsyD, and Ruth Yáñez, MSW, Todd Molfenter, Ph.D.

## References

[1] Kannarkat JT, Smith NN, McLeod-Bryant SA. Mobilization of telepsychiatry in response to COVID-19-moving toward 21^st^ century access to care. Adm Policy Ment Health. 2020;47(4):489–491. 32333227 10.1007/s10488-020-01044-z PMC7180652

[2] Wind TR, Rijkeboer M, Andersson G, Riper H. The COVID-19 pandemic: the ‘black swan’ for mental health care and a turning point for e-health. Internet Interv. 2020;20:100317. 32289019 10.1016/j.invent.2020.100317 PMC7104190

[3] Knopf A. Addiction telemedicine comes into its own with COVID‐19. Alcohol Drug Abuse Wkly. 2020;32(13):5–6.

[4] Druss BG. Addressing the COVID-19 pandemic in populations with serious mental illness. JAMA Psychiatry. 2020;77(9):891–892. 32242888 10.1001/jamapsychiatry.2020.0894

[5] Samuels EA, Clark SA, Wunsch C, et al. Innovation during COVID-19: improving addiction treatment access. J Addict Med. 2020;14(4):e8–e9. 32404652 10.1097/ADM.0000000000000685 PMC7236851

[6] Kreek MJ, LaForge KS, Butelman E. Pharmacotherapy of addictions. Nat Rev Drug Discov. 2002;1(9):710–726. 12209151 10.1038/nrd897

[7] Ban TA. Pharmacotherapy of mental illness – a historical analysis. Prog Neuropsychopharmacol Biol Psychiatry. 2001;25(4):709–727. 11383974 10.1016/s0278-5846(01)00160-9

[8] McCance-Katz EF. *SAMHSA/HHS: An Update on the Opioid Crisis*. Rockville, MD: Substance Abuse and Mental Health Services Administration; 2018. Available at https://www.samhsa.gov/sites/default/files/aatod_2018_final.pdf. 10.1176/appi.ps.201800281 30099944

[9] Becker S, Chaple M, Freese T, et al. Virtual reality for behavioral health workforce development in the era of COVID-19. J Subst Abuse Treat. 2020;121:108157. 33223379 10.1016/j.jsat.2020.108157 PMC7545203

[10] Fixsen DL, Blasé KA, Naoom SF, Wallace F. Core implementation components. Res Social Work Pract. 2009;19(5):531–540.

[11] Faria-Schützer DB, Surita FG, Alves VLP, Bastos RA, Campos CJG, Turato ER. Seven steps for qualitative treatment in health research: the Clinical-Qualitative Content Analysis. Cien Saude Colet. 2021;26(1):265–274. 33533847 10.1590/1413-81232020261.07622019

[12] Substance Abuse and Mental Health Services Administration. Using technology-based therapeutic tools in behavioral health services. In: Treatment Improvement Protocol (TIP) Series 60. HHS Publication No. (SMA) 15-4924. Rockville, MD: Substance Abuse and Mental Health Services Administration; 2015. 26889536

[13] Ellimoottil C, An L, Moyer M, Sossong S, Hollander JE. Challenges and opportunities faced by large health systems implementing telehealth. Health Affairs. 2018;37(12):1955–1959. 30633667 10.1377/hlthaff.2018.05099

[14] Blandford A, Wesson J, Amalberti R, AlHazme R, Allwihan R. Opportunities and challenges for telehealth within, and beyond, a pandemic. Lancet Glob Health. 2020;8(11):e1364–e1365. 32791119 10.1016/S2214-109X(20)30362-4 PMC7417162

[15] Greenberg AJ, Haney D, Blake KD, Moser RP, Hesse BW. Differences in access to and use of electronic personal health information between rural and urban residents in the United States. J Rural Health. 2018;34(suppl 1):s30–s38. 28075508 10.1111/jrh.12228 PMC5505819

[16] Molfenter T, Roget N, Chaple M, et al. Use of telehealth in substance use disorder services during and after COVID-19: online survey study. JMIR Ment Health. 2021;8(2):e25835. 33481760 10.2196/25835 PMC7895293

[17] Centers for Disease Control and Prevention. Increase in fatal drug overdoses across the United States driven by synthetic opioids before and during the COVID-19 pandemic. *CDC Health Alert Netw*o*rk*. 2020.

[18] Pfefferbaum B, North CS. Mental Health and the Covid-19 Pandemic. N Engl J Med. 2020;383(6):510–512. 32283003 10.1056/NEJMp2008017

[19] Weine S, Kohrt BA, Collins PY, et al. Justice for George Floyd and a reckoning for global mental health. Glob Ment Health. 2020;7:e22. 10.1017/gmh.2020.17 PMC7490771 32963794

[20] Ayanian JZ, Buntin MB. In pursuit of a deeper understanding of racial justice and health equity. JAMA Health Forum. 2020;1(6):e200765. 10.1001/jamahealthforum.2020.0765 36218519

